# Socio-cultural features and help-seeking preferences for leprosy and turbeculosis: a cultural epidemiological study in a tribal district of Maharashtra, India

**DOI:** 10.1186/s40249-015-0064-y

**Published:** 2015-07-28

**Authors:** Amar Prakash Maske, Pravin Arun Sawant, Saju Joseph, Uma Satish Mahajan, Abhay Machindra Kudale

**Affiliations:** The Maharashtra Association of Anthropological Sciences, Centre for Health Research and Development (MAAS-CHRD), 201, Akanksha Residency, B-Wing, Second Floor, Near Shivaji Statue, Aundh Gaon, Pune, 411007 Maharashtra India; Savitribai Phule Pune University, Ganeshkhind, Pune, Maharashtra 411007 India

**Keywords:** Socio-cultural features, Help seeking, Leprosy, Tuberculosis, Tribal, Cultural epidemiology, ACSM, IEC, India

## Abstract

**Background:**

India is a major contributor to the global burden of leprosy and tuberculosis (TB), which adversely affects the poorest tribal communities. Despite prioritisation by disease control programmes, programme performance for leprosy and TB in tribal communities continues to be a challenge. In addition to access to services and infrastructural limitations, socio-cultural concepts of illness causation and related help seeking (HS) rooted in distinct features of tribal culture need to be addressed to improve programme outcomes.

**Methods:**

A cultural epidemiological survey of leprosy and TB patients was carried out using a locally adapted, semi-structured explanatory model interviews. A total of 100 leprosy and 50 TB patients registered for treatment at government health facilities were selected randomly from tribal dominant blocks of the Thane district, Maharashtra state. The perceived causes (PCs) of leprosy and TB in patients were compared based on prominence categories. The relationship between PCs as predictors, and disease conditions and HS preferences as outcome variables were assessed using multivariate logistic regression.

**Results:**

In the multivariate logistic regression model with disease conditions as outcome variables, TB patients were significantly more likely to report PCs in the categories of ingestion; health, illness and injury; and traditional, cultural and supernatural. Tuberculosis patients more frequently first sought help from private facilities as compared to leprosy patients who preferred government health facilities. In a combined analysis of leprosy and TB patients employing multivariate logistic regression, it was found that patients who reported PCs in the environmental and contact-related categories were more likely to visit traditional rather than non-traditional practitioners. In another multivariate combined model, it was found that patients who reported PCs in the traditional, cultural and supernatural category were significantly more likely to visit private rather than public health facilities.

**Conclusion:**

Cultural concepts about illness causation and associated HS behaviours should be considered as priorities for action, which in turn would provide the necessary impetus to ensure that tribal patients seek help in a timely and appropriate manner, and could facilitate improvement in programme performance in general.

**Electronic supplementary material:**

The online version of this article (doi:10.1186/s40249-015-0064-y) contains supplementary material, which is available to authorized users.

## Multilingual abstracts

Please see Additional file 1 for translations of the abstract into the six official working languages of the United Nations.

## Background

Tuberculosis (TB) and leprosy often occur together, and leprosy usually only occurs in places where TB occurs. Countries such as India, Brazil, Indonesia, Bangladesh, Democratic Republic of the Congo, Nepal and Myanmar report large numbers of both TB and leprosy cases [1]. In 2012, there were approximately 233,000 new cases of leprosy worldwide and nearly all of them were from countries where TB is endemic [2].

India has the highest number of leprosy cases in the world [3, 4]. In 2013, of the total 215,656 new leprosy cases detected worldwide, India accounted for 126,913 [4]. Despite integration of leprosy services with general health care (2002–3) and making leprosy diagnosis and treatment services available free of charge at all primary health centres (PHCs) across India, and even after the attainment of the goal of leprosy elimination as a public health problem in 2005, the number of new leprosy cases continue to increase, with the disease prevalent with moderate endemicity in about 15 % of Indian districts [5, 6].

Of the globally estimated nine million TB cases in 2013, 24 % of the TB cases occurred in India, making it the country with the world’s highest TB burden [7]. The Revised National Tuberculosis Control Programme (RNTCP), based on the directly observed treatment, short-course (DOTS) strategy, has been implemented in India through the general health system across 692 districts and 35 states and union territories under the umbrella of the National Health Mission [8]. The RNTCP implements all the components of the World Health Organization’s (WHO’s) Stop TB Strategy, and has made great strides in achieving global targets for new smear positive case detection of 70 % and treatment success of 85 %. Despite massive efforts from the RNTCP, TB continues to remain a major public health problem in India, with an estimated 2.2 million incident cases reported in 2013 alone [7].

Leprosy and TB have medical and social consequences in India that mainly affect segments of the population living in poor socio-economic conditions [9–12]. In India, tribal people live in geographical isolation, mostly in remote, inaccessible hilly areas. Tribal people have distinct cultures and want to retain their cultural identity while at the same time attain economic development [13]. They are referred to as backward, based on their apparent lack of capacity to benefit from available opportunities for development, which makes them a notably vulnerable segment of the population [13]. Reducing the prevalence and improving control of leprosy and TB among tribal populations remain priorities for leprosy as well as TB programmes [14–16]. This priority is reflected through the Indian central government’s commitment of full (100 %) assistance for detection and treatment of leprosy cases, especially for the entire tribal population, and full (100 %) central assistance for ensuring the supply of anti-TB drugs and equipment in tribal areas [17]. The information, education and communication (IEC) plan of the National Leprosy Eradication Programme (NLEP 2012–17) identifies tribal communities as important priority groups [16]. In the RNTCP under the National Strategic Plan for TB Control (2012–17), the social action plan for marginalised and vulnerable communities also includes a designated tribal action plan [18].

Both leprosy and TB share priorities concerning needs for specific resources and expertise for timely diagnosis and initiating treatment. Socio-cultural concepts of illness regarding leprosy and TB are important as they can result in delays in seeking appropriate diagnosis and treatment, and in turn affect the effectiveness of public health programmes and illness outcomes [19–22]. Research studies documented that delay in presentation to a health facility supposedly on account of socio-cultural beliefs about illness causation contributes to delays in initiating TB and leprosy treatment [20, 23, 24].

Furthermore, various research studies carried out in India focused either on socio-cultural factors or on help-seeking (HS) preferences for leprosy or TB independently without considering their relationship [20, 21, 25–28]. The few studies that have considered socio-cultural factors associated with TB and HS behaviours of TB patients were undertaken in non-tribal areas [29, 30]. In the context of co-existence of both leprosy and TB in tribal dominant communities, there have been no attempts to identify the common and disease-specific socio-cultural features of both these diseases, specifically to distinguish cultural features that apply for control of either both diseases, or which may be relevant for the control of one of the diseases. These integrated studies are essential to strengthen the ongoing national programme agenda and initiatives to achieve goals of leprosy- and TB-free India.

Further, such integrated research studies require consideration of how cultural concepts of leprosy and TB illness affect HS preferences and practices of patients. To achieve this, two steps are essential: first, it is necessary to identify social and cultural features of these illnesses, and second, it is important to examine how these features influence HS preferences. In this paper, we have compared socio-cultural features of leprosy and TB revealed through perceived causes (PCs) and their associated HS preferences and practices amongst leprosy and TB patients. In consonance with the above mentioned steps, this paper aims to: (i) compare common and distinctive PCs of leprosy and TB as reported by patients, (ii) present and compare first help-seeking (FHS) practices for both leprosy and TB patients, and (iii) analyse how PCs are related to FHS practices.

## Methods

### Study area

**Fig. 1:**
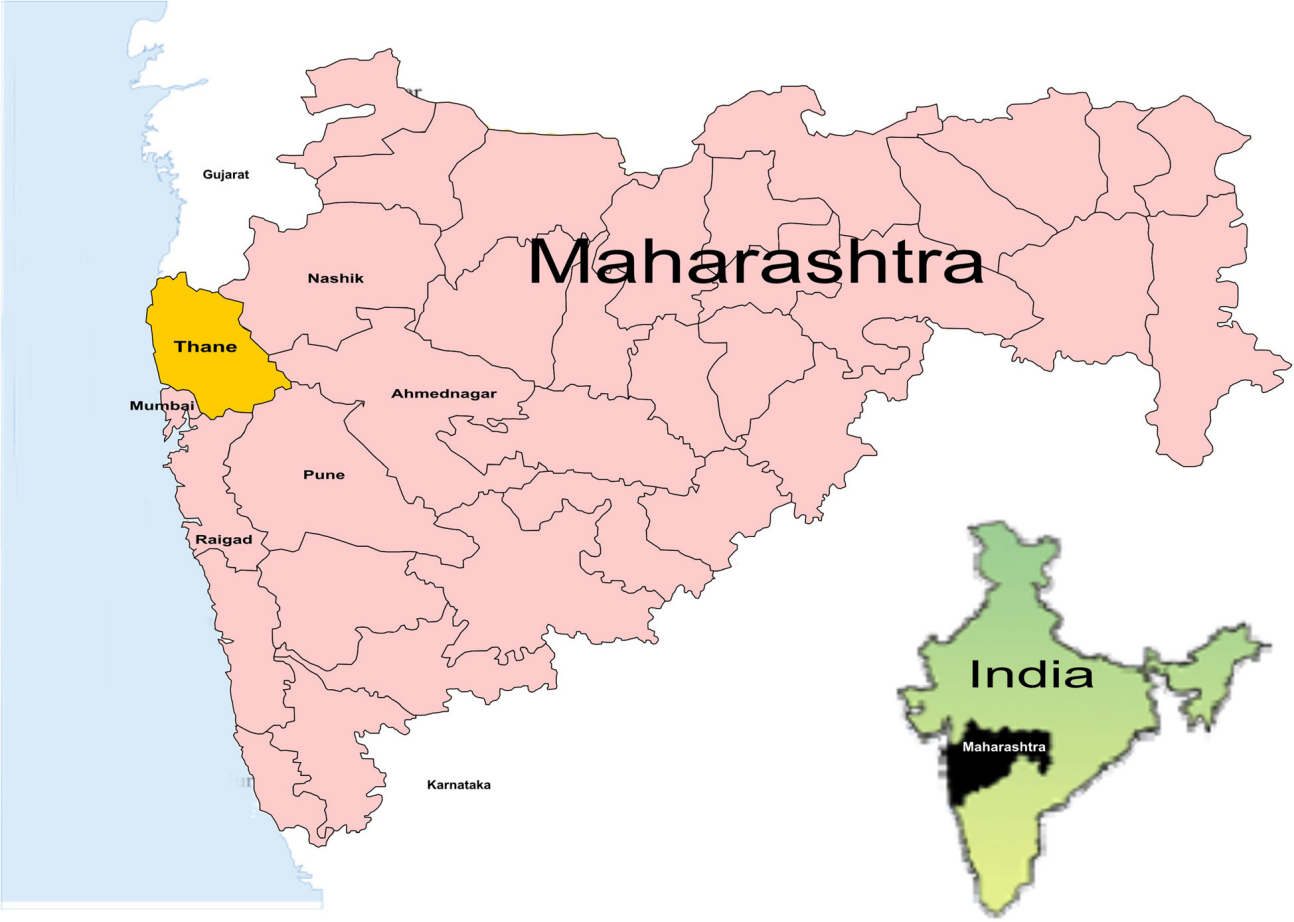
Map of Maharashtra State in India showing Thane district

The study was conducted in the Thane district, Maharashtra state. Thane is the most populous district in the country [31]. A total of 63 % of the rural population residing in nine blocks of the district are predominantly tribal [32]. In 2012–13, the district reported the highest number of active leprosy cases and TB cases for Maharashtra, with 2963 newly detected leprosy cases out of a total 18,715 cases in the state [33, 34], and 9933 TB cases registered for treatment out of a total 137,237 registered TB patients in the state [8] (see Fig. 1).

### Study groups/sample selection

Leprosy and TB patients registered for treatment at PHCs constituted the study group. The study was implemented in four randomly selected tribal blocks. A total of 18 PHCs from the four blocks provided the sampling frame. From the treatment registers maintained at the PHCs and TB units, a master list of 473 leprosy and 842 TB patients registered for treatment between April 2011 and September 2012 was prepared. As per the generic protocol^1^ from the master list, 100 leprosy and 50 TB patients were randomly selected and interviewed to detect a difference of about 20 % in the presence or absence of a cultural explanatory variable with 80 % power and 95 % confidence interval (CI). To achieve this level of statistical power and CI, fewer patients are required for a reference point other than 50 % other patients in the comparison group. Previous experience has shown that this sample is adequate to detect socio-cultural and HS behaviours of interest [35].

### Data collection/research tools

A cultural epidemiological survey of leprosy and TB patients was carried out using a locally adapted, semi-structured and pilot-tested explanatory interview model based on the framework of the Explanatory Model Interview Catalogue (EMIC) [36]. The model was focused on culturally relevant features of illness experiences (patterns of distress), their meanings (PCs) and related behaviours (HS). The cultural epidemiological approach and EMIC have been developed in a broad range of studies of tropical diseases including onchodermatitis [37], malaria [38], leprosy [39] and TB [29, 35].

Using the EMIC, the cultural explanatory variables of PCs were grouped into the following categories: ingestion; health, illness and injury; environmental; traditional, cultural and supernatural; contact-related; and miscellaneous. The ingestion category used for inquiry in the EMIC included factors such as food, water, smoking, alcohol and prescribed medicine. The health, illness and injury category included injury, accident, surgery, insect bite, physical exertion, blood problems, prior illness, neglect of prior illness, anatomical or physical problems, constitutional weakness, heredity and mental emotional stress. The environmental category included sanitation; personal hygiene; germs that cause infection; heat, cold or humoral; and climate and seasonal change. The traditional, cultural and supernatural category included contamination/contact (ritual pollution); sexual pollution; punishment for prior deeds; demons, fate, gods, stars and karma; evil eye and sorcery. The contact-related category included close contact with someone with the same problem and sexual contact. The miscellaneous category included other factors, or ones that the participants either didn’t know about or didn’t want to divulge.

Government health staff at the PHCs and local health workers at the village level facilitated the survey. The study respondents were contacted for interviews with the help of an accredited social health activist (ASHA) – a grass-root level health provider – from their respective villages. Technical officers, who had post-graduate degrees in social sciences and public health and were well versed in the local Marathi language, were trained in quantitative and qualitative data collection in a 2-week training programme, which was followed by pilot testing. The interviews were recorded using digital audio recorders to avoid loss of qualitative data collected during the interviews. One of the investigators interviewed the patient and the other noted and recorded the patient’s responses.

### Data analysis

The categorical and numerical data from the EMIC interviews were verified by double entry, cleaned and analysed using Epi Info™ software (version 3.5.3). SPSS for Windows (version 16.0) was the statistical software used for the advanced analysis. We examined the frequencies of spontaneous and probed coded cultural epidemiological variables representing categories of PCs and HS, considering healthcare providers in the public sector either private or traditional. The analysis computed the prominence of PCs and, for the analysis of association, timely appropriate HS categories. To specify the relative prominence of each category of leprosy and TB illness experience, meaning and behaviour, spontaneous responses to open-ended questions were assigned a prominence score of 2, a prominence score of 1 if mentioned only after probing, and a prominence score of 0 if not mentioned at all. The single most important PC contributed an additional value of three, yielding a total prominence score of 0 to 5 for each category. The scores were compared for leprosy and TB patients using the Mann-Whitney *U*-test to identify significant differences, if any, in PCs between leprosy and TB (disease conditions) and FHS preferences. To facilitate this analysis, the FHS variable was categorised into two sets: first, into non-traditional and traditional practitioners, and second, into the public and private sector. The non-traditional practitioners category included local health workers; PHCs or sub-centres; government hospitals; block level primary health centre (BPHC); community health centres (CHCs); health camps; pharmacists; Ayurveda, Unani, Siddha and homeopathy; private allopathic doctors; private allopathic specialists; and non-governmental organisation (NGO) clinics, private hospitals and nursing homes. The traditional practitioners category included local herbal healers; faith healers; and healing temples, Dargah or church.

The public sector category included local health workers; PHCs or sub-centres; government hospitals; BPHC; CHCs; and health camps. The private sector category included pharmacists; Ayurveda, Unani, Siddha and homeopathy; private allopathic doctors; private allopathic specialists; and NGO clinics, private hospitals and nursing homes, plus the traditional practitioners.

Both sets of FHS categories served as the outcome variables and helped identify the role of predictors, which included socio-demographic characteristics and PCs. The chi-square test of independence was applied to identify significant differences, if any, between socio-demographic characteristics, disease conditions and FHS preferences. Further, the chi-square test was also applied to identify the significant differences, if any, between disease conditions and FHS preferences.

Based on these univariate analyses, multivariate logistic regression was performed to study associations between PCs with the conditions of leprosy and TB. A multivariate logistic regression analysis was performed for disease conditions as outcome variables, with leprosy as a reference disease category. The PCs, which were significantly associated with the disease conditions of leprosy and TB in univariate analyses, were entered one by one into the multivariate logistic regression model. For each PC, ‘not reported’ was considered a reference category. Perceived causes with *p*-value ≤ 0.05 were retained in the multivariate logistic regression model, and further adjusted for socio-demographic variables such as age, gender, literacy, marital status, occupation and tribal/non-tribal community. Three multivariate models were constructed: first considering the disease condition as the outcome variable, second considering non-traditional practitioners as FHS preferences versus traditional practitioners as an outcome variable, and third considering the public sector as FHS preferences versus the private sector as an outcome variable. Model reports adjusted odds ratios (AORs), their 95 % CIs, *p*-values and Nagelkerke R^2^.

Open-ended data from the interviews were translated into English, entered into a word processor (Microsoft Word) and imported in a template format that allows pre-structured coding by interview item in MAXQDA (version 11), a software programme for qualitative data management. The coded data were analysed to clarify aspects of illness-related meanings and HS behaviours. Variables of interest were imported into MAXQDA as selection variables. Key selection variables such as PCs and HS with higher prominence scores were imported from the quantitative dataset to select records of particular interest. Qualitative thematic analysis clarified the nature and meanings of the coded variables and their relationship to FHS preferences.

### Ethical considerations

This study was approved by the Institutional Ethics Committee of the Maharashtra Association of Anthropological Sciences (MAAS). Written consent was obtained from the respondents after they were explained the purpose of the study. Interviews were conducted in local languages and at places convenient for the respondents to ensure privacy. Pseudonyms were used in data and text to protect the identity of respondents.

## Results

### Socio-demographic characteristics of patients

The proportion of women was higher in the leprosy sample compared to the TB sample, but in general, gender was not significantly associated across disease conditions. Literacy levels show significant differences between patient groups: more TB patients were literate compared to leprosy patients (*p* = 0.011). Both leprosy and TB patients were primarily engaged in agricultural activities, or working as unskilled or skilled labourers (see Table 1).

**Table 1:** Socio-demographic characteristics of respondents by disease conditions, FHS-Non-traditional vs Traditional and FHS-Public vs Private

		Disease condition			FHS - Non traditional vs Traditional			FHS - Public vs Private		
Characteristics	No. of respondents	Leprosy (*n* = 100) (%)	TB(*n* = 50) (%)	*p* - value	Non - traditonal (*n* = 135) (%)	Traditonal (*n* = 15) (%)	*p* - value	Public (*n* = 91) (%)	Private (*n* = 59)(%)	*p* - value
**Age (in years)**				0.908			0.744			0.481
<=32	76	67.1	32.9		90.8	9.2		57.9	42.1	
>32	74	66.2	33.8		89.2	10.8		63.5	36.5	
**Gender**				0.246			0.512			0.275
Male	68	61.8	38.2		88.2	11.8		55.9	44.1	
Female	82	70.7	29.3		91.5	8.5		64.6	35.4	
**Education**				**0.011**			0.744			0.333
Illiterate	76	76.3	23.7		90.8	9.2		64.5	35.5	
Literate	74	56.8	43.2		89.2	10.8		56.8	43.2	
**Material Status**				0.765			0.806			0.734
Unmarried	17	58.8	41.2		94.1	5.9		52.9	47.1	
Married	121	67.8	32.2		89.3	10.7		61.2	38.8	
Widowed/Divorced/Separated/Cohabiting	12	66.7	33.3		91.7	8.3		66.7	33.3	
**Occupation**					0.135		0.561			0.292
Labourer	60	70.0	30.0		88.3	11.7		58.3	41.7	
Cultivator/Land Owner	59	71.2	28.8		93.2	6.8		67.8	32.2	
Others	31	51.6	48.4		87.1	12.9		51.6	48.4	
**Tribal Status**				**0.015**			0.461			0.204
Tribal	131	70.2	29.8		89.3	10.7		62.6	37.4	
Nontribal	19	42.1	57.9		94.7	5.3		47.4	52.6	
**Disease condition**							**0.001**			**<0.0001**
Leprosy	100				96.0	4.0		75.0	25.0	
TB	50				78.0	22.0		32.0	68.0	

*Test applied – Chi-square test of independence

### Perceived causes (PCs) of leprosy and TB

A higher number of leprosy patients compared to TB patients reported being unaware of the cause of their illness (leprosy 28/100, 28 %; TB 5/50, 10 %, *p* < 0.05). Consider the following accounts:

*“Now I can’t say anything. I didn’t understand anything. I just thought that there is a patch. But I didn’t know how it was caused. Later, when I had itching, I asked a lady how her patch went away. She told me that I have to take tablets and only then it will go.”* (Female leprosy patient, married, 28 years old, literate, unskilled labourer)

*“[My TB] might have been caused because of others who have the illness. I might have gotten it while I was walking outdoors.”* (Female TB patient, unmarried, 21 years old, literate, student) (see Table 2).

**Table 2:** Mean prominence scores of perceived causes reported by disease conditions, FHS-Non-traditional vs Traditional and FHS-Public vs Private

	Disease condition – Leprosy vs TB			FHS - Non-traditional vs. Traditional			FHS - Private vs. Public		
Perceived Causes	Leprosy (*n* = 100)	TB (*n* = 50)	*p*-value	Non-traditional (*n* = 35)	Traditional (*n* = 15)	*p*-value	Public (*n* = 91)	Private (*n* = 59)	*p*-value
**Ingestion**	**0.69**	**2.88**	**<0.0001**	**1.36**	**1.93**	0.178	**1.01**	**2.05**	**0.015**
Food	0.10	0.64	**0.001**	0.27	0.40	0.187	0.22	0.37	0.122
Water	0.33	0.36	0.908	0.36	0.20	0.967	0.35	0.32	0.862
Smoking	0.06	0.64	**<0.0001**	0.25	0.27	0.597	0.14	0.42	**0.006**
Alcohol	0.12	0.50	0.109	0.22	0.47	0.222	0.13	0.42	0.131
Prescribe medicine	0.02	0.10	0.987	0.01	0.33	0.167	0.02	0.08	0.843
**Health Illness or Injury**	**2.31**	**2.06**	0.974	**2.20**	**2.47**	0.639	**2.30**	**2.12**	0.793
Injury, accident, surgery	0.21	0.08	0.505	0.14	0.40	**0.023**	0.09	0.29	**0.005**
Insect Bite (Mosquitoes, flies etc.)	0.09	0.02	0.375	0.07	0.07	0.586	0.09	0.03	0.751
Physical exertion/work	0.39	0.32	0.612	0.38	0.27	0.922	0.35	0.39	0.161
Blood problems	0.69	0.30	**0.046**	0.61	0.13	0.067	0.75	0.27	**0.008**
Prior illness	0.09	0.14	0.985	0.10	0.13	0.220	0.13	0.07	0.776
Neglect of prior illness	0.17	0.06	0.311	0.13	0.20	0.146	0.15	0.10	0.813
Anatomical or physical problem	0.25	0.20	0.837	0.21	0.47	0.362	0.23	0.24	0.986
Constitutional weakness	0.23	0.56	**0.029**	0.30	0.67	0.070	0.26	0.46	0.573
Hereditary	0.15	0.24	0.064	0.19	0.07	0.811	0.19	0.17	0.469
Mental Emotional Stress	0.04	0.14	0.296	0.07	0.07	0.815	0.05	0.10	0.933
**Environmental**	**0.79**	**1.22**	**0.007**	**0.87**	**1.53**	**0.030**	**0.87**	**1.03**	0.143
Sanitation	0.19	0.06	0.185	0.16	0.00	0.160	0.19	0.08	0.463
Personal Hygiene	0.03	0.12	**0.029**	0.06	0.07	0.909	0.02	0.12	**0.015**
Germs or infection	0.24	0.32	0.126	0.24	0.47	0.565	0.23	0.32	0.363
Heat-/Cold, humoral	0.15	0.24	0.315	0.14	0.53	**0.020**	0.15	0.22	0.402
Climate or seasonal change	0.18	0.48	**0.002**	0.26	0.47	**0.007**	0.27	0.29	0.539
**Traditional Cultural Supernatural**	**1.12**	**1.46**	**0.041**	**1.17**	**1.80**	0.082	**1.07**	**1.49**	**0.019**
Contamination/contact (ritual pollution)	0.01	0.00	0.480	0.01	0.00	0.739	0.01	0.00	0.421
Sexual pollution	0.06	0.04	0.758	0.04	0.20	**<0.0001**	0.05	0.05	0.347
Punishment for prior deed	0.18	0.34	**0.001**	0.21	0.40	0.114	0.20	0.29	**0.039**
Demons, fate, God, stars, karma	0.35	0.82	**0.006**	0.50	0.53	0.452	0.37	0.71	**0.013**
Evil eye, sorcery etc.	0.52	0.26	0.392	0.41	0.67	**0.021**	0.43	0.44	0.217
**Contact-related**	**0.60**	**0.92**	**0.207**	**0.61**	**1.60**	**0.009**	**0.65**	**0.80**	0.354
Close contact with someone with the same problem	0.59	0.80	0.340	0.57	1.47	**0.010**	0.65	0.68	0.543
Sexual contact	0.01	0.12	0.215	0.04	0.13	**0.001**	0.00	0.12	**0.030**
**Miscellaneous**	**2.00**	**1.34**	**0.145**	**1.85**	**1.13**	**0.217**	**1.76**	**1.81**	**0.775**
Other	0.73	0.92	0.184	0.79	0.80	0.965	0.74	0.88	0.490
Cannot say/no idea	1.27	0.42	**0.009**	1.06	0.33	0.134	1.02	0.93	0.827

*Test applied – Mann–Whitney *U* test

### Ingestion-related causes

Overall, ingestion-related causes were more prominently reported by TB patients (*p* < 0.0001). Tuberculosis patients were significantly more likely to report food (leprosy 8/100, 8 %; TB 13/50, 26 %, *p* = *0.001*) and smoking (leprosy 3/100, 3 %; TB 14/50, 28 %, *p* < *0.0001*) as the PCs of TB, as illustrated by the quotes below:

*“It [TB] might have been caused by the food. Vegetables and all…as we put fertilisers to the vegetables, they might have caused the illness.”* (Male TB patient, married, 40 years old, illiterate, construction worker)

*“The illness increased because of bidi [crude cigarette]. While doing labour on the truck, I used to smoke bidi, so this illness might have been caused by that.”* (Male TB patient, married, 40 years, literate, unemployed)

Ingestion-related causes such as water, alcohol and prescribed medicine had no distinct qualitative differences in responses among leprosy and TB patients. The following accounts illustrate this:

*“While in the field, we just drink whatever water there is. Who has checked it? But yes, I feel that it [leprosy] might have been caused by the water.”* (Female leprosy patient, married, 42 years old, literate, cultivator/landowner)

*“Yes, because of the drinking water. If a person with TB is around or consuming the water we drink or eating leftover food, it [TB] may have been caused by that.”* (Male TB patient, married, 35 years old, illiterate, unskilled labourer)

As compared to patients who first sought help from the public sector, the patients who first sought help from the private sector significantly reported smoking as the cause of their illness (*p* = 0.006). Here’s one such account:

*I think mostly it is due to tobacco. I have been chewing tobacco since I was 12 years old {…}* I continued to chew tobacco even after marriage. *After breakfast in the morning, in the afternoon after lunch and in the evening after dinner, I eat tobacco.”* (Male TB patient, married, 26 years old, literate, agricultural labourer)

### Health, illness and injury causes

At group level, health, illness and injury causes had no significant differences in mean prominence values in leprosy or TB patients. However, leprosy patients were more likely to report blood problems (leprosy 39/100, 39 %; TB 12/50, 24 %, *p* < *0.05*) as the cause:

*“I must have some problem in my blood; that’s what’s caused it.”* (Female leprosy patient, married, 43 years old, literate, housewife)

*“The doctor always asked me to test my blood, so I feel that this problem [leprosy] must have occurred due to a problem in my blood.”* (Female leprosy patient, married, 54 years, literate, housewife)

Tuberculosis patients were more likely to report constitutional weakness (leprosy 22/100, 22 %; TB 19/50, 38 %, *p* < *0.05*) as the cause of their illness:

*“Yes, I used to think so. I feel that I have been weak since childhood. I often have pain in my hands and legs.”* (Female TB patient, married, 24 years old, literate, agricultural labourer)

Other health, illness and injury causes such as insect bites, physical exertion/work, prior illness, neglect of prior illness, anatomical or physical problems, heredity and mental/emotional stress had no distinct qualitative differences in responses of leprosy and TB patients. The following accounts illustrate this:

*“I feel so, but how will I be able to eat without doing any work? I think I have it [leprosy] because of the work. Yes I think so.”* (Female leprosy patient, widowed, 50 years old, illiterate, unskilled labourer)

*“I must have got the illness due to overworking. We have to do a lot of hard work and that’s why I think I suffer from this illness. * (Female TB patient, married, 28 years old, illiterate, cultivator/landowner)

Injury as the cause was more reported by patients who first visited traditional providers (*p* < 0.05) as opposed to non-traditional providers.

*“I fell off a bicycle and had a lot of injuries. The wounds became white. I think it [leprosy] might have been caused because of that.”* (Male leprosy patient, married, 25 years old, illiterate, unskilled labourer)

### Environmental causes

Environmental causes were more prominently reported by TB patients (*p* = 0.007). Lack of personal hygiene (leprosy 3/100, 3 %; TB 6/50, 12 %, *p* < *0.05*) was significantly more reported by TB patients:

*“At work, we get very limited time for lunch, just half an hour. So we only wash our hands lightly and then immediately eat. So, I have a suspicion that it [TB] might have been caused by that.”* (Male TB patient, married, 24 years old, literate, cultivator/ landowner)

Climate change as the cause was more significantly reported by TB patients (*p* < 0.05) and also by patients who visited traditional providers for FHS, as the following attest:

*“It [TB] might have been caused by going from here and there. We have to work in filth. In different places, the climate is different and we have to work in those [environments]. [But] if I don’t move around, what would my family eat? I think about that. I work in filth, it stinks, and that’s why it (TB) might have been caused.”* (Male TB patient, married, 52 years old, literate, skilled labourer)

*“In my opinion, this illness was caused because of the weather and it’s gotten worse because of the food. If you have a tobacco chewing habit or any other addiction, then it gets even worse. I am more sure that it is caused by the weather…”* (Male TB patient, married, 35 years, literate, agricultural labourer)

Environmental causes such as sanitation; germs/infection; heat, cold or humoral; and climate and seasonal changes had no distinct qualitative differences in responses of leprosy and TB patients. The following quotes illustrate this:

*“It might have happened because I came into contact with germs”.* (Male leprosy patient, married, 35 years old, literate, unskilled labourer)

*“Might have been caused because germs might have entered my mouth.”*

(Male TB patient, married, 26 years old, literate, unskilled labourer)

### Traditional, cultural and supernatural causes

Traditional, cultural and supernatural causes were more prominently reported by TB patients (*p* = 0.041). Causes related to punishment for prior deeds (leprosy 11/100, 11 %; TB 17/50, 34 %, *p* < *0.001*); and demons, fate, gods, stars and karma (leprosy 30/100, 30 %; TB 26/50, 52 %, *p* < *0.001*) were significantly associated with TB patients. The following accounts illustrate this:

*“That must be my fate, that’s why it happened to me. I think it’s to settle prior deeds. What to do if I have bad fate?”* (Female TB patient, married, 23 years old, literate, agricultural labourer)

*“I dont believe in stars and planets (astrology), but whatever is given in our fate that tends to happen. So, I think its my fate thats why it (TB) caused me.”* (Female TB patient, married, 30 years old, literate, housewife)

*“People keep saying that these are my sufferings. You might have kicked someone, so you might have their curse. I kicked my mother, she cursed me [your arms and feet will fall off]…such thoughts used to come to my mind.”* (Male leprosy patient, married, 58 years old, literate, unemployed)

Other traditional, cultural and supernatural causes such as contamination/contact, sexual pollution and evil eye, sorcery had no qualitative differences in leprosy and TB patients. The following illustrate this:

*“They perform karni [sorcery]. It is due to karni that I have fallen ill.”* (Male TB patient, married, 50 years old, literate, unskilled labourer)

*“Yes, that happens. If somebody is insane, they may do such things [sorcery]. I even feel that it [leprosy] may have been caused by that.”* (Female leprosy patient, widowed, 50 years old, illiterate, unskilled labourer)

### Contact-related causes

Both leprosy and TB patients reported close contact with someone with the same illness as the cause of their illness (leprosy 24/100, 24 %; TB 15/50, 30 %), as illustrated by the following accounts:

*“I think some friends of mine may have it [leprosy]. I might have touched them and then acquired it myself. One of my friends at work has it [leprosy]. My feet or hands might have touched him. We used to take paint from the same bucket. Just by dipping my hand in that [bucket], I may have got it [leprosy].”* (Male leprosy patient, married, 21 years old, literate, painter on construction site)

Sexual contact as the cause of illness was more prominently reported by TB patients and significantly more by patients who first visited traditional providers (*p* < 0.001). However, responses were not elaborated on, as the below attests to:

*“Yes, I think so. Because of sexual pollution, I might have got the disease.”*

(Female TB patient, separated, 22 years old, illiterate, cultivator/landowner)

### Multivariate logistic regression by disease conditions: leprosy versus TB

Adjusted analysis with socio-demographic characteristics and explanatory variables of PCs are shown in Table 3.

**Table 3:** Multivariate logistic regression by disease conditions – Leprosy vs TB

Variables	Disease condition (Leprosy vs TB)		
	AOR	95 % CI	*p*-value
Constant	1.222		0.859
**Age**			
<=32 years	Reference		
>32 years	2.643	0.832–8.394	0.099
**Gender**			
Female	Reference		
Male	1.480	0.494–4.438	0.484
**Literacy**			
Literate	Reference		
Illiterate	0.313	0.103–0.953	**0.041**
**Marital status**			
Widowed/Divorced/Separated/Cohabiting	Reference		
Unmarried	0.218	0.021–2.249	0.201
Married	0.538	0.100–2.900	0.471
**Occupation**			
Other	Reference		
Labourer	0.197	0.050–0.768	**0.019**
Cultivator/Land owner	0.218	0.054–0.872	**0.031**
**Tribal community**			
Non-tribal	Reference		
Tribal	0.601	0.142–2.535	0.488
**Perceived causes**			
Food	8.079	2.408–27.112	**0.001**
Smoking	3.752	1.595–8.828	**0.002**
Anatomical or physical problem	0.096	0.019–0.470	**0.004**
Constitutional weakness	5.579	2.131–14.608	**<0.0001**
Demons, fate, God, stars, karma	3.258	1.776–5.976	**<0.0001**
Evil eye, sorcery etc.	0.424	0.182–0.984	**0.046**

Multivariate logistic regression model: outcome variable—disease condition Leprosy vs TB, where Leprosy is reference category, for Perceived causes—Not reported is reference category, R^2^ = 0.568

In the multivariate logistic regression model, with disease conditions as outcome variables, TB patients were five times more likely to report PCs such as food (AOR = 8.079, 95 % CI = 2.408–27.112) and constitutional weakness (AOR = 5.579, 95 % CI = 2.131–14.608) than leprosy patients (*p* < 0.001). Smoking (AOR = 3.752, 95 % CI = 1.595–8.828) and demons, fate, gods, stars and karma (AOR = 3.258, 95 % CI = 1.776–5.976) were three times more likely to be reported by TB patients (*p* < 0.001) as the PCs. Anatomical or physical problems and evil eye, sorcery, etc. were significantly more likely to be reported by leprosy patients.

### Help-seeking (HS) preferences of leprosy and TB patients

Government health facilities were the first preferred stop for three-quarters of the leprosy patients, whereas just one-third of the TB patients approached government health facilities first (*p* < 0.0001). Tuberculosis patients were more likely to seek help first from private providers (*p* = 0.002) or traditional providers (*p* < *0.001*) (see Table 4). Patients’ HS preferences are summarised by the following accounts:

**Table 4:** Percent reported first help seeking by disease conditions: Leprosy and TB

First help seeking preferences	Disease condition – Leprosy or TB		
	Leprosy (*n* = 100)	TB (n = 50)	*p*-value
**FHS-Public**	75.0	32.0	**<0.0001**
Local Health Worker	9.0	0.0	**0.029**
Primary Health Centre or Sub-Centre	51.0	22.0	**0.001**
Government Hospital, BPHC, CHC	14.0	10.0	0.487
Health Camp	1.0	0.0	0.478
**FHS - Private**	21.0	46.0	**0.002**
Druggist or Pharmacy for Advice	1.0	4.0	0.216
Ayurveda, Unani, Siddha, Homeopathy	1.0	0.0	0.478
Private Allopathy Doctor	13.0	34.0	**0.002**
Private Allopathic Specialist	0.0	4.0	**0.044**
NGO clinic, Private Hospital or Nursing Home	6.0	4.0	0.607
**FHS - Traditional**	4.0	22.0	**0.001**
Local Herbal Healer	0.0	2.0	0.156
Faith Healer	3.0	20.0	**<0.0001**
Healing Temple, Dharga or Church	1.0	0.0	0.478

*Test applied Chi-square test of independence applied for first help seeking and disease condition. Percentages are calculated for combined spontaneous and probed responses

*“It’s better in the government hospital. I thought it would be better if I get tablets there [sub-centre of PHC], so I went there.”* (Female leprosy patient, married, 40 years old, illiterate, agricultural and brick kilns labourer)

*“I knew the doctor [private], so I went to him. If I don’t have money, he treats me on credit. So I went to him two to three times. Once, I couldn’t pay but he still gave me injection and tablets…so I only went to him.”* (Male TB patient, married, 24 years old, literate, cultivator/landowner)

*“I thought it would be someone from the family, someone might have done karni [sorcery]. Women amongst us do that and a bhagat [traditional healer] can break the spell.”* (Male leprosy patient, married, 58 years old, literate, construction worker)

*“My cough got worse and because of that, my food consumption gradually reduced. My energy gradually reduced. So my parents told me that some outside energy might be acting and that’s why I have TB, so I went to a bhagat.”* (Female TB patient, separated, 22 years old, illiterate, cultivator/landowner)

Among the government health facilities, PHCs or sub-centres were prominently preferred as FHS sources by leprosy patients (*p* < 0.001). Some of the reasons are outlined below:

*“In the private clinic, one has to pay money. Government clinics are for the poor. Our people [tribal] go to government clinics [PHC] for any [health] problem.”* (Female leprosy patient, married, 30 years old, literate, unskilled labourer)

*“The [free] tablets are available only at the government hospital. They are available at other places [with a cost], but I went only to the government clinic [to avail it free]. My daughter was malnourished so I used to go there and get many things for her [for free] over there.”* (Female leprosy patient, married, 25 years old, illiterate, tenant cultivator)

Among traditional providers, faith healers were more preferred as FHS sources by TB patients (*p* < 0.0001). Their reasons are described below:

*“Initially, family members thought that something might have been done so we went there. My husband and mother-in-law felt that some karni might have been performed.”* (Female TB patient, married, 23 years old, literate, worker at a company)

*“I thought somebody has done something. Such severe problems had never occurred before, so how come they happened all of a sudden. So I went to a bhagat.”* (Male, TB patient, married, 52 years, literate, worker at a company)

### Effects of PCs of illness on FHS preferences

In univariate analyses (see Table 2), patients who reported heat, cold or humoral (mean prominence = 0.53, *p* = 0.020), sexual pollution (mean prominence = 0.20, *p* < 0.0001), evil eye, sorcery (mean prominence = 0.67, *p* = 0.021), close contact with someone with the same problem (mean prominence = 1.47, *p* = 0.010) or sexual contact (mean prominence = 0.13, *p* = 0.001) as the PCs of their illness were more likely to prefer traditional providers for FHS.

Patients who reported smoking (mean prominence = 0.42, *p* = 0.006); injury, accident and surgery (mean prominence = 0.29, *p* = 0.005); personal hygiene (mean prominence = 0.12, *p* = 0.015); punishment for prior deeds (mean prominence = 0.29, *p* = 0.039); demons, fate, gods, stars or karma (mean prominence = 0.71, *p* = 0.013); and sexual contact (mean prominence = 0.12, *p* = 0.030) as the PCs of their illness were more likely to prefer the private sector for FHS. Patients who reported blood problems (mean prominence = 0.75, *p* = 0.008) as their PC of illness were more likely to prefer the public sector for FHS (see Table 2).

### All patients

As per the multivariate logistic regression shown in Table 5, all patients who reported heat, cold or humoral as their PC of illness were three times more likely to prefer traditional providers for FHS than non-traditional providers (AOR = 2.818, 95 % CI = 1.213–6.548, *p* = 0.016). Patients who reported close contact with someone with the same problem as the PC of their illness were about 1.5 times more likely to prefer traditional providers for FHS (AOR = 1.444, 95 % CI = 1.056–1.975, *p* = 0.021) (see Table 5).

**Table 5:** Multivariate logistic regression: First Help Seeking—Non-traditional vs Traditional

Variables	FHS—Non-traditional vs Traditional		
	AOR	95 % CI	*p*-value
Constant	0.049		0.076
**Age**			
<=32 years	Reference		
>32 years	1.248	0.354–4.397	0.730
**Gender**			
Female	Reference		
Male	1.125	0.284–4.458	0.867
**Literacy**			
Literate	Reference		
Illiterate	0.718	0.186–2.771	0.631
**Marital status**			
Widowed/ divorced/ separate	Reference		
Unmarried	0.152	0.004–5.461	0.302
Married	0.993	0.098–10.092	0.995
**Occupation**			
Other	Reference		
Labourer	0.415	0.081–2.125	0.291
Cultivator/Land owner	0.243	0.046–1.293	0.097
**Tribal community**			
Non-tribal	Reference		
Tribal	3.578	0.319–40.082	0.301
**Perceived causes**			
Heat-/cold, humoral	2.818	1.213–6.548	**0.016**
Close contact with someone with the same problem	1.444	1.056–1.975	**0.021**

Multivariate logistic regression model: outcome variable—FHS Non-traditional vs Traditional, where Non-traditional is reference category, for perceived causes—not reported is reference category, R^2^ = 0.186

Patients who perceived demons, fate, gods, stars or karma as the cause of their illness were 1.6 times more likely to prefer the private sector for FHS (AOR = 1.620, 95 % CI = 1.056–2.487, *p* = 0.027), as shown in Table 6.

**Table 6:** Multivariate logistic regression: First Help Seeking—Public vs Private Sector

Variables	FHS—Public Vs Private		
	AOR	95 % CI	*p*-value
Constant	1.069		0.939
**Age**			
<=32 years	Reference		
>32 years	0.689	0.322–1.472	0.336
**Gender**			
Female	Reference		
Male	1.583	0.730–3.434	0.245
**Literacy**			
Literate	Reference		
Illiterate	1.045	0.465–2.350	0.915
**Marital status**			
Widowed/ divorced/ separate	Reference		
Unmarried	1.010	0.187–5.457	0.991
Married	0.957	0.252–3.635	0.948
**Occupation**			
Other	Reference		
Labourer	0.748	0.285–1.964	0.556
Cultivator/Land owner	0.545	0.207–1.435	0.219
**Tribal community**			
Non-tribal	Reference		
Tribal	0.615	0.217–1.747	0.362
**Perceived causes**			
Demons, Fate, God, Stars, Karma	1.620	1.056–2.487	**0.027**

Multivariate logistic regression model: outcome variable—FHS Public vs Private, where Public is reference category, for perceived causes—Not reported is reference category, R^2^ = 0.095

## Discussion

Studies on leprosy and TB have stressed that a lack of knowledge about the causes, modes of transmission and treatment affects not just HS behaviours of patients, but also programme control strategy [40–45]. Despite the advances in the treatment of leprosy and TB, the findings of this study documented various socio-cultural beliefs of illness causation which are prevalent in tribal areas of Maharashtra; some of these beliefs are common for both leprosy and TB, while others are distinct. Leprosy and TB patients attributed significance to socio-cultural beliefs in the categories of ingestion; health, illness and injury; environmental; and traditional, cultural and supernatural. These findings are similar to studies done by Weiss *et al.* [35] and Vidhani and Vadgama [46] in urban Tamil Nadu and rural Gujarat, respectively, and are consistent with studies conducted by Singh et al. [47] in urban Chandigarh and Singh [48] in rural Madhya Pradesh. Studies conducted in tribal areas of Madhya Pradesh identified seeking help from traditional healers for diagnosis to be embedded in the socio-cultural fabric of perceived illness causation, such as punishment for past sins [49, 50]. Atre et al. [30] in their study of leprosy patients in rural Maharashtra found that traditional beliefs were still prevalent amongst patients, which in turn influenced their HS behaviours. Findings of this study suggest that both TB and leprosy patients, irrespective of the reported causes, did seek care from traditional sector providers, further validating the fact that HS in tribal communities is greatly influenced by people’s socio-cultural belief systems.

Study findings also stressed on the inadequate knowledge (biomedical information) about illness causation that led patients to seek treatment from traditional health providers, thus further delaying appropriate diagnosis and treatment. Tuberculosis patients significantly reported FHS from private providers compared to leprosy patients who preferred government health facilities. These findings are consistent with other studies conducted among TB patients in India and elsewhere [51, 52]. These studies showed that TB patients, before presenting to public health centres, visited private practitioners where diagnosis is often inadequate, thus delaying TB diagnosis and treatment [51, 52]. The study conducted in rural Maharashtra documented that leprosy patients sought help from private providers and traditional healers [53].

The IEC activities under the leprosy control programme have had limited impact and in the post-integration era,^2^ emphasis on IEC has gotten further diluted [44, 54]. The multivariate logistic regression model employed in this study demonstrated that patients who reported environmental and contact-related causes made a significant contribution to outcomes and availed traditional sector providers. In another multivariate model, patients who reported traditional, cultural and supernatural causes were significantly more likely to visit private practitioners than public health facilities. Though leprosy and TB are both curable, these socio-cultural beliefs and their influence on HS preferences question the performance of the present IEC campaign for a leprosy-free India initiated under NLEP and the advocacy, communication and social mobilisation (ACSM) under RNTCP. More so, the ACSM under RNTCP needs to take cognisance of these socio-cultural beliefs, as our study documented that fewer tribal TB patients went to government health facilities for FHS, preferring to go to traditional healthcare providers.

These findings implied that the present IEC campaign for leprosy and TB-ACSM activities needs to be promoted in tribal dominated areas in the form of intensified health education and public awareness campaigns to increase awareness on the causes, transmission and availability of government health facilities for leprosy and TB. Both leprosy and TB programmes should prepare IEC materials that take cognisance of social and cultural features and HS preferences identified by tribal respondents, and try to incorporate culture-friendly, gender-sensitive and programme-appropriate messages in the local tribal dialect.

Under the RNTCP, case detection depends on a patient’s ability to self-identify symptoms of TB and voluntary reporting at a health facility for diagnosis [35, 55]. Although the social action plan prepared by the RNTCP in 2013 [56] acknowledged three major groups of barriers, namely socio-cultural, economic and health system, in the implementation of RNTCP in tribal areas, the plan did not spell out how the gap between traditional and biomedical knowledge could be filled. Cultural meaning and concepts about illness causation and documented HS behaviours for TB as shown in this study are not thus far considered as the priority action domains or action points under the RNTCP. In view of this, inclusion of these priority action domains and points into periodic programme evaluation and planning institutional and implementation arrangements to increase access to and utilisation of treatment services for tribal communities would provide necessary impetus to improve timely and appropriate HS among tribal patients, which would in turn contribute to the overall improvement in programme performance [22, 57].

Early reporting and registration for the treatment of leprosy in tribal areas is socially driven and depends on the tribal people’s knowledge about leprosy and its consequences [28]. If control programmes expect suspects to avail treatment facilities in order to reduce prevalence, delay and transmission, then efforts should be made to widely increase awareness among people that leprosy and TB treatment is provided free of charge and available at government health facilities. In addition, public-private mix (PPM) initiatives, which at present are considered only for TB under the RNTCP, should take cognisance of the presence of traditional sector providers in tribal areas, with the PPM initiatives also extended to them. Such PPM initiatives could be initially started with the TB programme, which might ensure continuity of care of TB patients availing treatment at private and traditional sector providers and would thereby avoid delays in initiation of TB treatment under the RNTCP. Inclusion of NGOs, traditional providers and private providers in health service delivery in tribal and inaccessible areas could enhance the reach of the RNTCP, and would further help improve TB programme performance.

Under the IEC plan for a leprosy-free India and TB-ACSM programmes, tribal patients should be educated on the importance of promptly seeking early diagnosis and treatment in order to promote self-reporting and early detection of hidden cases. In the prevailing context of co-existence, if the documented socio-cultural concepts and meanings among the tribal population about leprosy and TB are not addressed in time, then increased availability of treatment facilities may not translate into an appropriate increase in utilisation of services [58, 59]. These socio-cultural features and associated preferences need to be better addressed by incorporating them into tribal-centric IEC leprosy-free India plans and TB-ACSM programme activities under the present leprosy and TB control programmes.

### Limitations of the study

This study, due to administrative constraints with regards to time, budgetary considerations and the generic and multi-centric nature of the protocol, could not recruit equal numbers of TB and leprosy patients. This study was carried out in government health facilities and both leprosy and TB patients were recruited only when they sought treatment in these facilities. This means that this study didn’t account for the perspectives of leprosy and TB patients who primarily sought services from private health sector facilities.

## Conclusion

Although identifying and discerning socio-cultural beliefs about leprosy and TB regarding illness causation and exploring associated HS preferences presents a difficult challenge for research, our study has identified the critical influence of traditional, cultural and supernatural beliefs in relation to seeking help from traditional sector providers for both leprosy and TB. Acknowledging the co-existence of both the disease conditions and in view of the need for convergent actions, these findings highlight the importance of preparing public-private-traditional sector mix models for TB and leprosy control to ensure continuity of care of tribal patients in order to avoid delays in early diagnosis and treatment initiation for both diseases. The cultural epidemiological approach used in this study is also likely to be useful for explaining other priority issues of TB and leprosy control, such as concepts of cure and stigma determining treatment adherence, socio-cultural determinants of default, drug reactions and drug resistance.

## Additional file

Additional file 1:
**Translation of the abstract into the six offical working languages of the United Nations.**

## Abbreviations

ACSM: Advocacy communication and social mobilisation
ASHA: Accredited social health activist
AOR: Adjusted odds ratio
BPHC: Block level primary health centre
CHC: Community health centre
CI: Confidence interval
DOTS: Directly observed treatment short-course
EMIC: Explanatory model interview catalogue
HS: Help seeking / help-seeking
IEC: Information education and communication
MAAS: Maharashtra association of anthropological sciences
NGO: Non-governmental organisation
NHM: National health mission
NLEP: National leprosy eradication programme
PC: Perceived cause
PHC: Primary health centre
PPM: Public-private mix
RNTCP: Revised national tuberculosis programme
TB: Tuberculosis
WHO: World health organization

## Footnotes

1 This study was a part of a multi-centric study led by the National Institute of Epidemiology (NIE) and initiated by a taskforce project of leprosy based at the Indian Council of Medical Research, New Delhi. The generic protocol was initiated and led by the NIE team and the MAAS-CHRD team, along with four other participating collaborators. The total sample size for leprosy was 600 (100 per site) and for TB it was 300 (50 per site).

2 Post-integration: In 2005, India achieved a leprosy prevalence rate <1/10000, and hence eliminated leprosy as a public health problem at the national level. With this achievement, the need for a separate vertical leprosy programme was diminished, and the Government of India initiated the second phase of the NLEP programme and integrated leprosy services into general health services after 2005.
