# Supplementary material for: Socio-cultural features and help-seeking preferences for leprosy and turbeculosis: a cultural epidemiological study in a tribal district of Maharashtra, India
Source: Infect Dis Poverty. 2015 Jul 28;4:33. doi: 10.1186/s40249-015-0064-y (PMC4517489; doi:10.1186/s40249-015-0064-y)
Supplement: Additional file 1: — Translation of the abstract into the six offical working languages of the United Nations. [file 40249_2015_64_MOESM1_ESM.pdf]

Translation of the abstract into the six official working languages of the United Nations

الخصائص الاجتماعية والثقافية وتفضيلات طلب المساعدة لمرضى الجذام والسل: دراسة وبائية ثقافية في المنطقة القبلية في ولاية ماهاراشترا بالهند

عمار ماسكه وبرافين ساوانت وساجو جوزيف وأوما ماهاجان وأبهاي كوديل

ملخص

معلومات عامة: تُعد الهند مصدراً رئيسياً للعبء العالمي من مَرَضَّي الجذام والسل (TB)، وهو ما يؤثر سلباً على أفقر المجتمعات القبلية. على الرغم من تحديد الأولويات بواسطة برنامج مكافحة الأمراض، لا يزال أداء البرنامج بالنسبة لمَرَضَّي الجذام والسل يشكل تحدياً في المجتمعات القبلية. بالإضافة إلى الوصول إلى الخدمات ومحدودية البنية التحتية، فإنه من أجل تحسين نتائج البرنامج يجب معالجة المفاهيم الثقافية الاجتماعية المتعلقة بأسباب المرض وما يتعلق بها من طلب المساعدة (HS)، تلك المفاهيم المتجذرة في سمات بارزة للثقافة القبلية.

المناهج: تم اجراء استبيان وبائي ثقافي عن مرضى الجذام والسل عن طريق مقابلات ذات نموذج تفسيري شبه منظم تم تكييفه محلياً. تم اختيار اجمالي 100 مريض من مرضى الجذام و50 مريضاً من مرضى السل عشوائياً من أولئك المسجلين للعلاج لدى المنشآت الصحية الحكومية ويقطنون بالتجمعات السكنية ذات السيادة القبلية في مقاطعة ثين بولاية مهاراشترا. وتمت مقارنة الأسباب المُدركة (PCs) لدى مرضى الجذام والسل التي تؤدي إلى الإصابة بالمرض وذلك اعتماداً على التصنيفات البارزة. تم تقييم العلاقة ما بين الأسباب المدركة كمُنبِّئات والحالات المرضية وتفضيلات طلب المساعدة كمتغيرات للنتائج باستخدام الانحدار اللوجستي متعدد المتغيرات.

النتائج: في نموذج الانحدار اللوجستي متعدد المتغيرات مع الحالات المرضية كمتغيرات للنتائج كان الأكثر احتمالاً بشكل كبير هو قيام مرضى السل بالإبلاغ عن الأسباب المُدركة وفقاً لتصنيفات الابتلاع؛ الصحة والمرض والإصابة؛ والتقليدية والثقافية والخرافية. سعى مرضى السل بشكل أكثر تواتراً إلى طلب المساعدة من المنشآت الخاصة أولاً مقارنة بمرضى الجذام الذين فضلوا المنشآت الصحية الحكومية. وفي تحليل مشترك عن مرضى الجذام والسل باستخدام الانحدار اللوجستي متعدد المتغيرات تم اكتشاف أن المرضى الذين قاموا بالإبلاغ عن أسباب مُدركة وفقاً للتصنيفات البيئية والمتعلقة باللمس كانوا أكثر احتمالاً لزيارة ممارسي الطب التقليدي بدلاً من ممارسي الطب غير التقليدي. في نموذج آخر مشترك متعدد المتغيرات تم اكتشاف أن الأكثر احتمالاً بشكل كبير هو قيام المرضى الذين أبلغوا عن أسباب مُدركة وفقاً للتصنيفات التقليدية والثقافية والخرافية بزيارة المنشآت الصحية الخاصة بدلاً من العامة.

الاستنتاج: يجب النظر إلى المفاهيم الثقافية عن مسببات المرض وما يتعلق بها من سلوكيات طلب المساعدة كأولويات للعمل وهو ما سيوفر بدوره الزخم اللازم لضمان أن المرضى القبليين سوف يسعون نحو الحصول على المساعدة فى الوقت المناسب وبالطريقة المناسبة وهو ما يمكن أن ييسر من تحسين أداء البرنامج بشكل عام.

Translated from English version into Arabic by Ran Abdel Rahman, through

TRANSLATORS
WITHOUT BORDERS

麻风病和肺结核的社会文化特点和求助偏好——印度马哈拉施特拉部落地区的文化流行病学研究

Amar Maske, Pravin Sawant, Saju Joseph, Uma Mahajan and Abhay Kudale

摘要

引言：印度严重加剧了全球麻风病和肺结核的疾病负担，对最贫困的部落社区产生不利的影响。尽管疾控项目倾向于优先防控部落社区的麻风病和肺结核，但其效果并不理想。除了服务通道和基础设施的限制，也需阐明病因的社会文化概念和植根于鲜明部落文化特色的相关求助方案，以促成项目成果。

方法： 本研究采用地区适宜的半结构化解释模型访谈法对麻风病和肺结核患者进行了文化流行病学调查。从马哈拉施特拉周塔纳地区的部落统治街区随机挑选了 100 名麻风患者和 50 名肺结核患者，上述患者均在政府卫生机构登记治疗过。基于突出类别，比较了麻风病和肺结核的认知原因。运用多元 logistics 回归分析法评估了认知原因、病情与求医偏好之间的关系，其中，前者作为预测因素，后者作为结果变量。

结果：在以病情为结果变量的多元 logistic 回归模型中，多数肺结核患者倾向于把认知原因分类为：摄取；健康、疾病和伤害；传统的、文化的和超自然的。肺结核患者常去私立机构就诊，而麻风病患者多愿意去政府医疗机构。对麻风病和结核病患者

的多因素 Logistic 回归综合分析表明，环境和接触类病因患者倾向于拜访传统行医者而非现代化行医者。在另一种多因素综合模型中，发现大部分传统、文化和超自然类病因患者更可能访问私立医院，而非公共卫生设施。
结论：在行动前，应该优先考虑病因的文化概念和相关求助行为，提供必要的动力以便于部落患者及时求助，获得适当的治疗，从而促进项目总体职能的提升。

Translated from English version into Chinese by Chen Jin, edited by Yang Pin, through

Caractéristiques socio-culturelles et préférences en matière de recherche d'aide dans le cas de la lèpre et de la tuberculose : étude épidémiologique culturelle dans le district tribal de Maharastra en Inde.

Amar Maske, Pravin Sawant, Saju Joseph, Uma Mahajan and Abhay Kudale

RÉSUMÉ

Contexte: L'Inde porte une grande part du fardeau mondial de la lèpre et de la tuberculose, deux maladies qui pèsent lourdement sur les communautés tribales les plus pauvres. Malgré la priorité qui leur est donnée, les programmes de lutte contre la lèpre et la tuberculose donnent encore des résultats mitigés dans ces communautés tribales. Outre les difficultés d'accès aux services et les limites des infrastructures, il importe de résoudre aussi des questions socioculturelles liées aux notions des causes des maladies et à la recherche d'aide associée, ancrées dans les particularités distinctives de la culture tribale.
Méthodes: Une enquête épidémiologique culturelle auprès de patients lépreux ou tuberculeux a été menée en utilisant des modèles d'entretiens explicatifs semi-structurés et adaptés localement. Un total de 100 lépreux et 50 tuberculeux inscrits en traitement dans des établissements de santé publics ont été sélectionnés de façon aléatoire dans les composantes tribales prédominantes du district de Thane, dans l'état de Maharashtra. Les causes perçues par les patients de la lèpre et de la tuberculose ont classées par catégories d'importance. La relation entre ces causes perçues en tant que facteur prédictifs, d'une part, et d'autre part les conditions de la maladie et les préférences de recherche d'aide en tant que variables résultantes a été évaluée en utilisant une régression logistique multivariée.
Résultats : Dans le modèle de régression logistique multivariée utilisant les conditions de la maladie comme variable résultante, les tuberculeux étaient significativement plus susceptibles de rapporter des causes perçues dans les catégories liées à l'ingestion, à la santé, la maladie et aux blessure, et à des aspects traditionnels, culturels et surnaturels. Ils étaient plus nombreux à demander l'aide de structures privées que les lépreux, qui privilégiaient pour leur part les établissements de santé publics. Dans une analyse combinée des lépreux et des tuberculeux par régression logistique multivariée, nous avons découvert que les patients rapportant des causes perçues liées à l'environnement, d'une part, et aux contacts d'autre part étaient plus susceptibles de consulter des tradipraticiens que des praticiens non traditionnels. Dans un autre modèle combiné multivarié, nous avons constaté que les patients qui rapportaient des causes perçues d'ordre traditionnel, culturel et surnaturel étaient significativement plus nombreux à consulter des tradipraticiens que des établissements de soins publics.
Conclusion: Les concepts culturels relatifs aux causes des maladies et les comportements de demande d'aide associés doivent être considérés comme des leviers d'action prioritaires pour inciter les malades des tribus à demander de l'aide au bon moment et de façon appropriée. Leur utilisation pourrait contribuer à améliorer les résultats d'ensemble des programmes de lutte contre les maladies.

Translated from English version into French by Suzanne Assenat, through

Социально-культурные особенности и предпочтения в выборе помощи при заболевании проказой и туберкулезом: культурно-эпидемиологическое исследование в племенном районе штата Махараштра, Индия

Амар Маске, Правин Савант, Сажу Жозеф, Ума Махажан и Абхау Кудали

АННОТАЦИЯ

Контекст: На долю Индии приходится самая большая часть больных проказой и туберкулезом (ТБ) во всем мире. Эти заболевания в значительной степени затрагивают самые бедные племенные сообщества. Несмотря на то, что они являются приоритетными группами в рамках программ по контролю заболеваний, эффективность борьбы с проказой и ТБ в этнических общинах остается недостаточной. Для повышения данной эффективности необходимо принимать во внимание не только проблемы ограниченного доступа к медицинским услугам и инфраструктуре, но и социально-культурную интерпретацию причин заболевания и предпочтения при выборе помощи (ВП), которые тесно связаны с культурой племени.

Методы исследования: культурно-эпидемиологическое исследование больных проказой и ТБ было проведено при помощи адаптированных к местным особенностям, полуформализованных интервью для построения объяснительной модели. Всего 100 больных проказой и 50 больных ТБ, зарегистрированных на лечении в государственных здравоохранительных учреждениях, были выбраны методом случайного отбора в племенном районе округа Тхане, штата Махараштра. Интерпретация больными причин (ИП) проказы и ТБ были сравнены по ведущим признакам. Оценка соотношения между ИП как независимой переменной и условий протекания болезни, а также предпочтений при выборе помощи как зависимых переменных была проведена с использованием многомерной логистической регрессии.

Результаты: в модели многомерной логистической регрессии, где заболевание являлось зависимой переменной, больные ТБ значительно чаще относили ИП к таким категориям, как заражение через рот; здоровье, болезнь и телесное повреждение; традиционное, культурное и сверхъестественное. Больные туберкулезом изначально обращались за помощью в частные учреждения чаще, чем больные проказой, которые предпочитали государственные здравоохранительные учреждения. При комбинированном анализе больных проказой и туберкулезом на основе многомерной логистической регрессии было выявлено, что больные, которые относили ИП к категориям, связанным с окружающей средой или контактами, чаще обращались к традиционным, чем к нетрадиционным врачам. В другой комбинированной многомерной модели было выявлено, что пациенты, которые относили ИП к традиционным, культурным или сверхъестественным категориям, чаще обращались в частные, а не в государственные учреждения.

Заключение: действия по борьбе с ТБ и проказой должны быть в первую очередь направлены на культурное восприятие причин болезни и проистекающие из этого предпочтения ВП, так как это может способствовать более частому обращению больных из племенных общин за своевременной и компетентной помощью и, соответственно, улучшению эффективности медицинских программ в целом.

Translated from English version into Russian by Irina_Ka, through

Características socioculturales y preferencias en la búsqueda de asistencia para la lepra y la tuberculosis: un estudio cultural y epidemiológico en el distrito tribal de Maharashtra, India

Amar Maske, Pravin Sawant, Saju Joseph, Uma Mahajan y Abhay Kudale

RESUMEN

Antecedentes: India es uno de los principales contribuyentes a la carga mundial de lepra y tuberculosis (TB), lo que afecta de manera adversa a las comunidades tribales más pobres. A pesar de la priorización de los programas de control de enfermedades, el desempeño de los programas para lepra y TB en las comunidades tribales continúa siendo un desafío. Además del acceso a servicios y las limitaciones de infraestructura, para mejorar los resultados del programa se necesita abordar los conceptos socioculturales de causas de la enfermedad y búsqueda de asistencia, los cuales están arraigados en los distintos aspectos de la cultura tribal.

Métodos: Se llevó a cabo una encuesta cultural-epidemiológica de pacientes con lepra y tuberculosis. La misma utilizó modelos de entrevistas explicativas semi-estructuradas, adaptadas al entorno local. Se seleccionaron al azar un total de 100 pacientes con lepra y 50 pacientes con tuberculosis provenientes de secciones predominantemente tribales en el distrito de Thane, estado de Maharashtra, quienes se habían registrado para tratamiento en instituciones médicas del gobierno. Las causas percibidas de lepra y TB en los pacientes se compararon con base en las categorías de relevancia. Mediante una regresión logística multivariable se evaluó la relación entre las causas percibidas como indicadores y las condiciones de la enfermedad y las preferencias de búsqueda de asistencia como variables de resultados.

Resultados: En el modelo de regresión logística multivariable con las condiciones de enfermedad como variables de resultado, era mucho más probable que los pacientes con TB reportaran causas percibidas en categorías de ingestión; salud, enfermedad y lesiones; y tradicional, cultural y supernatural. Los pacientes con tuberculosis con mayor frecuencia primero buscaban asistencia en instituciones privadas en comparación con los pacientes con lepra, que preferían las instituciones médicas del gobierno. En un análisis combinado de pacientes con lepra y TB que utilizó regresión logística multivariable se encontró que era más factible que los pacientes que reportaban causas percibidas en el medio ambiente y categorías asociadas con el contacto, visitaran profesionales tradicionales en lugar de los no-tradicionales. En otro modelo multivariable combinado, se encontró que era significativamente mucho más probable que los pacientes que reportaban las causas percibidas en las categorías tradicional, cultural y supernatural, asistieran a instituciones de salud privadas que públicas.

Conclusión: Los conceptos culturales sobre las causas de enfermedad y los comportamientos de búsqueda de asistencia deberían considerarse una prioridad para la acción, lo que a su vez proporcionará el ímpetu necesario para asegurar que los pacientes tribales busquen asistencia de manera oportuna y adecuada, y podría facilitar una mejoría en el desempeño del programa en general.

Translated from English version into Spanish by Maria Alejandra Aguada, through

TRANSLATORS
WITHOUT BORDERS
